# A mutation in the *FZL* gene of *Arabidopsis* causing alteration in chloroplast morphology results in a lesion mimic phenotype

**DOI:** 10.1093/jxb/ert237

**Published:** 2013-08-19

**Authors:** Michela Landoni, Alessandra De Francesco, Silvia Bellatti, Massimo Delledonne, Alberto Ferrarini, Luca Venturini, Roberto Pilu, Monica Bononi, Chiara Tonelli

**Affiliations:** ^1^Dipartimento di Bioscienze, Università degli Studi di Milano, via Celoria 26, 20133 Milano, Italy; ^2^Dipartimento di Biotecnologie, Università degli Studi di Verona, Strada le Grazie 15, 37134 Verona, Italy; ^3^Dipartimento di Scienze Agrarie e Ambientali-Produzione, Territorio, Agroenergia, via Celoria 2, 20133 Milano, Italy

**Keywords:** *Arabidopsis thaliana*, chloroplast, double mutants, expression analysis, lesion mimic mutants (LMMs), reactive oxygen species (ROS)

## Abstract

Lesion mimic mutants (LMMs) are a class of mutants in which hypersensitive cell death and defence responses are constitutively activated in the absence of pathogen attack. Various signalling molecules, such as salicylic acid (SA), reactive oxygen species (ROS), nitric oxide (NO), Ca^2+^, ethylene, and jasmonate, are involved in the regulation of multiple pathways controlling hypersensitive response (HR) activation, and LMMs are considered useful tools to understand the role played by the key elements of the HR cell death signalling cascade. Here the characterization of an *Arabidopsis* LMM lacking the function of the *FZL* gene is reported. This gene encodes a membrane-remodelling GTPase playing an essential role in the determination of thylakoid and chloroplast morphology. The mutant displayed alteration in chloroplast number, size, and shape, and the typical characteristics of an LMM, namely development of chlorotic lesions on rosette leaves and constitutive expression of genetic and biochemical markers associated with defence responses. The chloroplasts are a major source of ROS, and the characterization of this mutant suggests that their accumulation, triggered by damage to the chloroplast membranes, is a signal sufficient to start the HR signalling cascade, thus confirming the central role of the chloroplast in HR activation.

## Introduction

Programmed cell death (PCD) is a metabolically active and genetically controlled process leading to cell death (Jones, 2001). In plants, this process occurs not only during normal development and senescence, but also during interactions with the environment in biotic and abiotic stress responses (van Doorn *et al*., 2011). One of the most studied forms of PCD is the cell death associated with the defence pathway known as the hypersensitive response (HR) (Lam *et al*., 2001)

When plants are attacked by pathogens, a first basal response, the so-called PAMP-triggered immunity (PTI) (Jones and Dangl, 2006), is activated after the recognition of conserved microbial/pathogen-associated molecular patterns (MAMPs/PAMPs). Pathogens can, however, overcome PTI by secreting effector proteins in the plant cell. In response, cells have developed specific receptors that recognize the effectors and activate a second layer of immunity, the effector-triggered immunity (ETI) (Jones and Dangl, 2006). ETI, a more rapid and robust defence response than PTI, is specifically set up when a pathogen *avr* (avirulence) gene is recognized by the complementary *R* (resistance) gene of the plant in a gene-for-gene interaction (Ellis *et al*., 2000), and the death of cells challenged by the pathogen (HR cell death) is often used as a barrier to limit pathogen growth in plant tissues (Tsuda and Katagiri, 2010). This response is also characterized by the activation of a complex defence system including the following: rapid ROS (reactive oxygen species) accumulation, production of defence molecules, cell wall strengthening, and activation of the expression of defence genes, with the aim of stopping not only pathogen diffusion but also further attacks by different pathogens (Muthamilarasan and Prasad, 2013). In particular, the so-called pathogen-related proteins (PRs) are a wide and heterogeneous group of proteins induced in cells under attack by pathogens, that have been demonstrated to act specifically in the control of pathogen infection (Van Loon *et al*., 2006).

The structure of the R protein appears to determine which are the positive regulators required for HR signalling: the TIR-NBS-LRR (Toll interleukin1 receptor–nucleotide-binding–leucine-rich repeat) class of R genes require *EDS1* (Enhanced Disease Susceptibility1) and *PAD4* (Phytoalexin Deficient4) genes, while the *R* genes encoding CC-NB-LRR (coiled-coil–nucleotide binding–leucine-rich repeat) proteins require the *NDR1* (Non-Race Specific Disease Resistance1) gene (Aarts *et al*., 1998; Lorrain *et al*., 2003).

The aberrant regulation of HR characterizes the lesion mimic mutants (LMMs), a group of mutants showing discrete leaf lesions and activation of defence responses in the absence of pathogen attack. This type of mutant thus appears to be a powerful tool to identify genes involved in the regulation of the cell death programme, to dissect the signalling pathways activated in this process, and to discover the cross-talk between them (Walbot *et al*., 1983; Greenberg and Ausubel, 1993; Dietrich *et al*., 1994; Greenberg *et al*., 1994; Lorrain *et al*., 2003).

The LMMs can be grouped into two classes: the initiation mutants, characterized by the presence of discrete lesions that identify functions related to the initiation phase of lesion formation; and the propagation mutants, characterized by the uncontrolled spread of the lesions and identifying functions related to the containment of HR cell death signalling (Lorrain *et al*., 2003).

The constitutive expression of biochemical and molecular markers associated with HR is one of the hallmarks of the LMMs: they are diagnostic of the activation of defence responses and give precise indications of which, among the different defence pathways, are specifically activated in the LMM analysed (Lorrain *et al*., 2003). In fact, several different signalling molecules are known to be involved in the HR pathway: salicylic acid (SA) (Gaffney, 1993; Devadas *et al*., 2002), ROS (Jabs *et al*., 1996; Levine *et al*., 1996; Zurbriggen *et al*., 2010), nitric oxide (NO) (Delledonne *et al*., 1998), ethylene, and jasmonate (JA) (Dong, 1998; Greenberg *et al*., 2000), and the activation of each of these pathways can be revealed by the analysis of specific markers.

The central role played by SA in plant–pathogen interactions has been demonstrated by the use of transgenic plants expressing the bacterial *NahG* gene (encoding salicylate hydroxylase) that were unable not only to accumulate SA but also to activate defence responses after pathogen attack (Gaffney *et al*., 1993). This evidence is supported by the higher level of SA generally found in LMMs in comparison with the wild type, and the suppression of lesion formation when different LMMs are crossed with *NahG* transgenic plants (Lorrain *et al*., 2003).

The characterization of the *sid2* (*salicylic acid induction deficient2*) mutant and its allelic *eds16* (*enhanced disease susceptibility16*), defective in SA biosynthesis and with enhanced susceptibility to pathogens, supported a central role for the enzyme isochorismate synthase, encoded by the *SID2* gene, in pathogen-stimulated SA biosynthesis (Wildermuth *et al*., 2001). The *EDS5* (*SID1*) gene is also involved in pathogen-mediated SA biosynthesis (Nawrath *et al*., 2002), and recently it has been suggested to be responsible for SA transport (Yamasaki *et al*., 2013).

The isolation of the *npr1* (*non expresser of PR genes1*) mutant, unable to activate *PR* gene expression, allowed the identification of the essential role played by *NPR1* in SA signalling, downstream of the *R* gene-mediated defence responses, but an *NPR1*-independent SA signalling pathway has also been reported (Shah, 2003; Dong, 2004).

Associated with hypersensitive cell death, pathogen attacks often trigger, in uninfected tissue, a sort of broad spectrum immunity to subsequent infections, called SAR (systemic acquired resistance) (Fu and Dong, 2013). The accumulation of the signalling molecule SA (Gaffney *et al*., 1993) and the expression of a group of disease-related genes, in particular *PR* genes (Van Loon *et al*., 2006), *GST* (glutathione *S*-transferase), *PRXc* (peroxidase C), and *Pal1* (phenylalanine ammonia lyase1) (Ward *et al*., 1991; Greenberg *et al*., 1994; Maleck *et al*., 2000), are known to be linked to the establishment of SAR.

The earlier signals generally reported to be connected with HR execution are the rapid rise of cytoplasmic Ca^2+^ and the production of ROS, generated not only at the cytoplasmic level, by the action of NADPH oxidases, but also in mitochondria and chloroplasts. In particular chloroplasts are thought to be the initial source of ROS immediately after pathogen recognition, thence this signalling is spread to the apoplast and then to the adjacent cells, leading to the selected death of the cells challenged by the pathogen (Zurbriggen *et al*., 2010).

In chloroplasts during HR, the generation of ROS can be the consequence of EEE (excess excitation energy); that is, photon intensity is in excess of that required for CO_2_ fixation, or can be the product of chlorophyll catabolism. This is supported by data reporting that mutations in genes involved in EEE dissipation (*LSD1*) or in chlorophyll catabolism (*ACD1* and *ACD2*) resulted in light-dependent lesion mimic phenotypes caused by photo-oxidative damage with formation of ROS (Mach *et al*., 2001; Pružinská *et al*., 2003; Mateo *et al*., 2004).

In this work, an *Arabidopsis* mutant with the typical appearance of the LMMs (i.e. characterized by the presence, early during development, of chlorotic lesions on rosette leaves, and the constitutive activation of defence responses) is described. Both lesion formation and defence response activation are SA dependent, requiring the functions of *EDS16*, *PAD4*, and *NPR1* genes, but are ethylene–JA independent.

Sequence analysis showed that the mutation was in the *At1g03160* gene encoding an FZO-like protein (FZL), playing a unique role in the determination of thylakoid and chloroplast morphology (Gao *et al*., 2006), and histological analysis confirmed the presence of chloroplasts with altered morphology in *fzl-*L*er* (mutation *fzl* in Landsberg *erecta* ecotype) mutants.

Data are presented showing that in the *fzl-Ler* mutant the loss of chloroplast integrity is linked to the activation of defence responses, and it is suggested that a chloroplast-generated signal plays a central role in the signalling cascade leading to defence activation and HR cell death.

## Materials and methods

### Plant material

The *fzl-*L*er* mutant was initially isolated during the generation of the transposant lines of the Exotic collection (http://Arabidopsis.info/CollectionInfo?id=31; last accessed 18 July 2013), ecotype Landsberg *erecta* (L*er*). Because, as shown in the Results, the *Ds* element did not co-segregate with the *fzl*-L*er* mutation, the characterization of this mutant was performed on the *fzl*-L*er* line obtained after the segregation of the *Ds* element.

The two T-DNA insertion lines of the Salk collection: Salk_033745 and Salk_009051 (provided by the NASC, Nottingham Arabidopsis Stock Centre, http://nasc.nott.ac.uk/) (Alonso *et al*., 2003) correspond to the *fzl* mutant in the Columbia ecotype previously characterized (Gao *et al*., 2006). In all the experiments, both the T-DNA insertional lines were used, always obtaining comparable results, so for this reason herein the *fzl*-Col mutant is referred to without further specifications.

The mutants *eds5*, *eds16*, *ein2*, *etr1*, *jar1*, *npr1*, *pad4*, and *vad1* were provided by the NASC.

### Plant growth conditions

*Arabidopsis thaliana* plants were grown in soil (Vegetal Radic, Tercomposti, Calvisano Brescia, Italy) in a greenhouse or *in vitro* in a growth chamber.

The seeds for *in vitro* growth were surface sterilized in 95% ethanol, soaked for 6min in 40% bleach, 0.1% Tween-20, and washed twice in sterile distilled water. The seeds were then sown in Murashige–Skoog medium (MS; SIGMA M-5524), supplemented with 0.7% Bacto agar (Difco) and 1% sucrose. The growth conditions in the greenhouse were 16h light (100µmol m^–2^ s^–1^ light intensity), 22 °C temperature, 60% humidity, while in the phytochamber (for *in vitro* growth) they were 16h light (100µmol m^–2^ s^–1^ light intensity), 22 °C temperature, and 40% humidity.

In the high temperature experiment, the temperature was 28 °C for the treatment, and 22 °C for the control; in the low light growth experiment, the light intensity was 50 µmol m^–2^ s^–1^ for the treatment and 100 µmol m^–2^ s^–1^ for the control.

### Genetic analysis

For double mutants analysis, *fzl-*L*er* plants, used as pollen donor, were crossed with the mutants *eds5*, *eds16*, *ein2*, *etr1*, *jar1*, *npr1*, *pad4* and *vad1*. The genotype of double mutants was determined by cleaved amplified polymorphic sequence (CAPS and dCAPS) analysis as described previously (Resnick *et al*., 2006; Stein *et al*., 2006).

The *fzl-*L*er* mutation was selected by CAPS analysis: using the primers EcoRVFor 5′-GAGCAACAACGTTGCCAAACAC-3′ and EcoRVRev 5′-ACTGCGATGGTAGAATTTTGAATTACTGA-3′, and the enzyme *Eco*RV, the wild-type DNA yielded a single band of 102bp, and the *fzl-*L*er* allele yielded two bands of 71bp and 31bp.

### Histochemistry

Callose and autofluorescence detection were performed as reported by Dietrich and colleagues (1994). Evan’s blue staining was performed as reported by Iriti and colleagues (2003). 3,3′-Diaminobenzidine (DAB) staining was performed as reported by Murgia and colleagues (2004).

### Cell death quantification

Cell death was quantified by electrolyte leakage measurement as previously reported (Roberts *et al*., 2013).

### H_2_O_2_ quantification

H_2_O_2_ was quantified as previously reported (Shi *et al*., 2013).

### Salicylic acid and salicylic acid glucoside measurement

Free and total SA were extracted and measured from 2g of dried tissue (3-week-old rosette), as previously described (Toiu *et al*., 2011).

### Chloroplast analysis

Chloroplasts of individual fixed mesophyll cells (Pyke and Leech, 1991) were observed using a Zeiss Axiophot D1 microscope equipped with differential interference contrast (DIC) optics.

### RNA isolation and expression analyses

The tissues were collected from wild-type and mutant plants, grown *in vitro* or in soil as specified in the different experiments.

The expression analyses were performed by the RT–PCR technique as previously reported (Landoni *et al*., 2010), or, in the case of double mutants, by real-time RT–PCR as previously reported (Lazzeri *et al*., 2012). The sequences of the oligonucleotides used are reported in Supplementary Table S1 available at *JXB* online for RT–PCR and in Supplementary Table S2 for real-time RT–PCR.

### Positional cloning of the fzl-Ler mutant

The mutant which was isolated was crossed to the Col ecotype, the F_1_ progeny (phenotype wild type). were allowed to self-fertilize, and in the F_2_ population the LMM phenotype, as expected, segregated 3:1 (1625:533, *P* > 0.70). DNA was collected from single F_2_ plants showing the LMM phenotype, from the F_1_ and from the parental lines, and positional cloning as reported by Lukowitz and colleagues (2000) was started. By the analysis of the collected recombinants with the Mapmaker program (Lander *et al*., 1987), it was found that the LMM mutation mapped within an interval of 5.4 cM flanked by the two SSLP (simple sequence length polymorphism) markers NF21B7 and ACC2 (TAIR SSLP collection: www.Arabidopsis.org).

### DNA library preparation and sequencing

DNA libraries with an insert size of 250bp were prepared starting from 10 µg of genomic DNA using a Paired-End DNA Sample Prep Kit (Illumina Inc., San Diego, CA, USA). Library quality control was performed with a High Sensitivity DNA Kit (Agilent, Wokingham, UK). Libraries were sequenced with an Illumina GAIIx sequencer (Illumina Inc., San Diego, CA, USA), and 75–100bp paired-end sequences were generated.

### Data analysis

The alignment against the reference genome (TAIR9 version of the *A. thaliana* genome) was performed with GenomeMapper v. 0.4, allowing for up to four mismatches and one gap in the reads. Data were processed using the SHORE v.0.4 pipeline (Ossowski *et al*., 2008). Trimmed reads shorter than 50bp were discarded. The variants were called with ShoreMap v. 1.1 in the genomic interval determined by positional cloning (Schneeberger *et al*., 2009). Known *Ler* variants were excluded from the analysis.

### Complementation of the fzl-Ler mutant

The 5900bp PCR-amplified fragment containing the *FZL* coding region and a sequence of ~1000bp 5′ of the ATG was amplified with the primers 3160-1F/1R using the Phusion™ DNA Polymerase (Fynnzymes) and cloned in the pCR-XL-TOPO vector (Invitrogen). The *Sac*I/*Xba*I fragment was then cloned in the binary vector PZP221 that was used to transform *fzl-*L*er* plants via *Agrobacterium tumefaciens* (strain GV3101) as previously reported (Clough and Bent, 1998). Transformed plants were selected on MS medium containing 100 µg ml^–1^ gentamycin sulphate.

## Results

### Isolation and phenotypic characterization of a new lesion mimic mutant

**Fig. 1.**
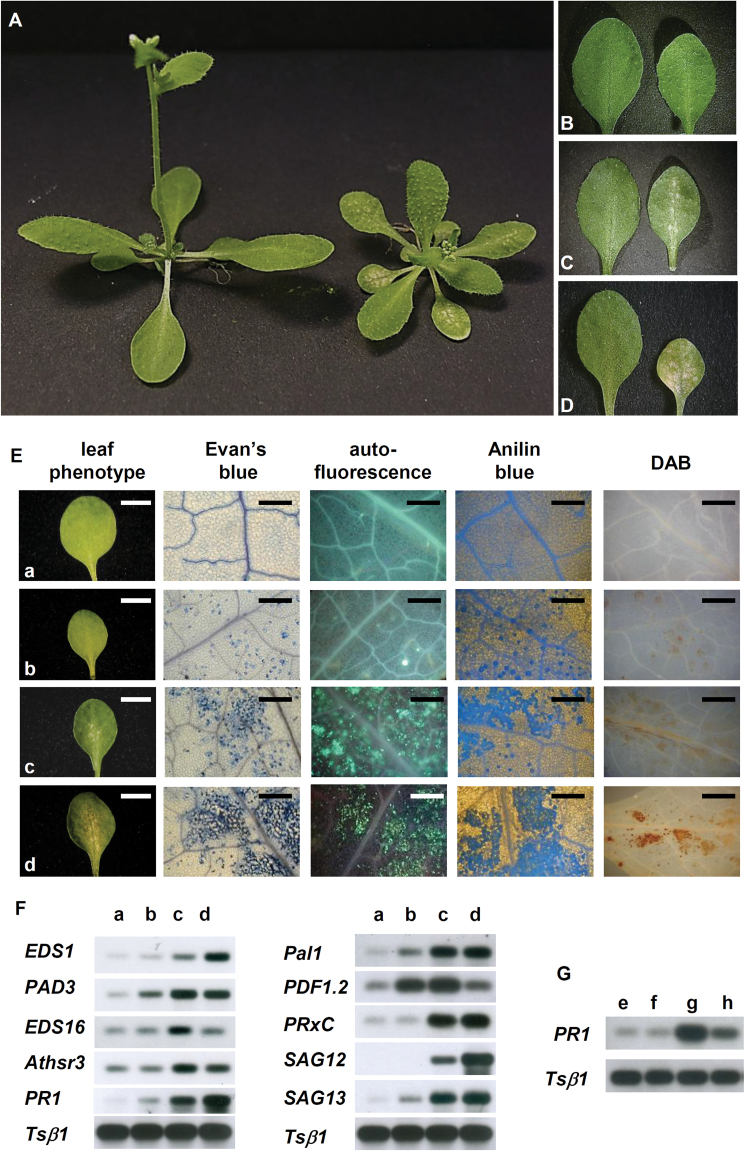
Phenotype of the mutant and analyses of the biochemical and molecular markers associated with defence responses. (A) Three-week-old wild-type (left) and mutant (right) plants. (B–D) Three-week-old wild-type (left) and mutant (right) leaves: while all the wild-type leaves are green, in the mutant the third pair of leaves are green (b, right), the second show small chlorotic spots (c, right), and the first show large chlorotic spots (d, right). (E) Histochemical analysis of 3-week-old wild-type and mutant leaves: in the first column is shown the phenotype of leaves analysed (a, wild-type green leaf; b, mutant green leaf; c, mutant leaf with small chlorotic spots; d, mutant leaf with large chlorotic spots), in the second column staining with Evan’s blue, in the third natural autofluorescence, in the fourth aniline blue staining, and in the fifth DAB staining. Bars indicate 5mm in the first column, 500 µm in the second, third, and fourth columns, and 1mm in the fifth column. (F) RT–PCR analysis of genes associated with plant defence responses in 3-week-old leaves of a wild-type and mutant plant (a, wild-type green leaf; b, mutant green leaf; c, mutant leaf with small chlorotic spots; d, mutant leaf with large chlorotic spots). (G) Analysis of SAR activation in the *fzl-*L*er* mutant by comparison of the expression levels of the *PR1* gene in wild-type rosette leaves (e), wild-type cauline leaves (f), mutant rosette leaves (g), and mutant cauline leaves (h).

During the generation of a gene-trapping collection of *A. thalian*a based on the *Ac/Ds* transposon system of maize (Sundaresan *et al*., 1995), a mutant showing chlorotic lesions on rosette leaves and reduced plant size was isolated (Fig. 1A). The lesions began to appear as small chlorotic spots close to the central vein of the rosette leaves, starting from 2–3 weeks after germination, then within a week to 10 d they enlarged to cover the entire leaf blade (Fig. 1B–D). The mutant seedlings also showed delayed development, with a marked reduction of the rosette size (Fig. 1A).

Taken together, the phenotypic alterations displayed by this mutant were reminiscent of the class of LMMs, characterized by the misregulation of the HR (Lorrain *et al*., 2003).

To test whether the mutant that was isolated could really be considered a member of this group of mutants, the presence of some biochemical markers that are known to be constitutively expressed in the LMMs was sought. In particular, the presence of callose, revealed by aniline blue staining, secondary metabolites, revealed by their natural fluorescence, H_2_O_2_ accumulation, revealed by DAB staining, and dead cells, revealed by Evan’s blue staining, was looked for.

These analyses were performed on 3-week-old rosette leaves of mutant plants that showed different degrees of lesion development (green leaves, leaves with small chlorotic spots, and leaves with large chlorotic spots) and on 3-week-old wild-type leaves (green) (Fig. 1E, first column).

In mutant plants, the biochemical markers analysed are present not only in and around the chlorotic lesions but also in completely green leaves (Fig. 1E, second row). In this tissue, however, although no chlorotic lesions were detected by macroscopic observation, the Evan’s blue staining revealed the presence of dead cells, singly or in small groups. The blue spots are localized close to the central vein, in the positions where the chlorotic lesions will subsequently appear during development of the mutant phenotype (Fig. 1E, second column). In wild-type leaves, neither dead cells nor other HR-specific biochemical markers were detected (Fig. 1E, first row).

The expression of a group of defence-related genes known to be generally constitutively expressed in LMMs was then analysed in mutant and in wild-type plants. For all the genes analysed, no or low expression was detected in wild-type leaves, while in mutant leaves the amount of these transcripts was higher than in the wild type, generally further increasing with the development of the lesions (Fig. 1F).

It was then checked whether, as observed for other LMMs (Dietrich *et al*., 1994; Devadas *et al*., 2002), *PR* genes, considered to be the executors of SAR (Fu and Dong, 2013), are also expressed in tissues that never develop lesions. The expression of *PR1* (Ward *et al*., 1991) was analysed in 3- to 4-week-old rosette leaves, that appeared completely green in wild-type plants and with chlorotic spots in mutants, and in cauline leaves, that appeared completely green in both wild-type and mutant plants.

While in wild-type rosette and cauline leaves, no PR1 expression was detected, in mutant leaves PR1 transcript is present not only in rosette leaves, but also in cauline leaves (Fig. 1G), even if the level of expression, quantified by real-time RT–PCR, is slightly lower than in green rosette leaves (data not shown). Further experiments will be needed to test the real activation of SAR in *fzl-*L*er* mutants.

These data indicated that the defence response pathway was constitutively activated in mutant plants, and, together with the histochemical data and the phenotypic traits previously described, suggested that the mutant which was isolated could be considered a propagative LMM.

### Genetic analysis and identification of the fzl-Ler mutation

The mutant, isolated as a homozygous recessive mutant, was crossed with its parental ecotype L*er*, and the F_1_ progeny, which showed a wild-type phenotype, were allowed to self-pollinate. The segregation of the mutant phenotype was analyzed in the F_2_ generation and a 3:1 ratio was observed (169:59, *P* > 0.9), as expected for a recessive mutation affecting a single locus.

The *Ds* association with the mutant phenotype was investigated by PCR amplification of the *GUS* (*β-glucuronidase*) reporter gene included in the *Ds* element (Sundaresan *et al*., 1995): an F_2_ population of 312 plants was analysed, and it was found that the mutation was not tagged by the *Ds* transposon, so positional cloning of this gene was set up. The mutant, in the original background (L*er*), was crossed to a Columbia wild-type plant and it was found that the mutant phenotype was still perfectly recognizable in the mixed background of the resulting F_2_. Therefore, 536 mutant plants were isolated from this segregant population and were analysed with SSLP molecular markers (Lukowitz *et al*., 2000).

The mutated locus was mapped in a 5.4 cM region flanked by the SSLP markers NF21B7 and ACC2 on chromosome 1. Further, SSLP/CAPS analysis failed to narrow down this genetic interval; therefore, a deep sequencing approach was used to isolate the mutation.

**Fig. 2.**
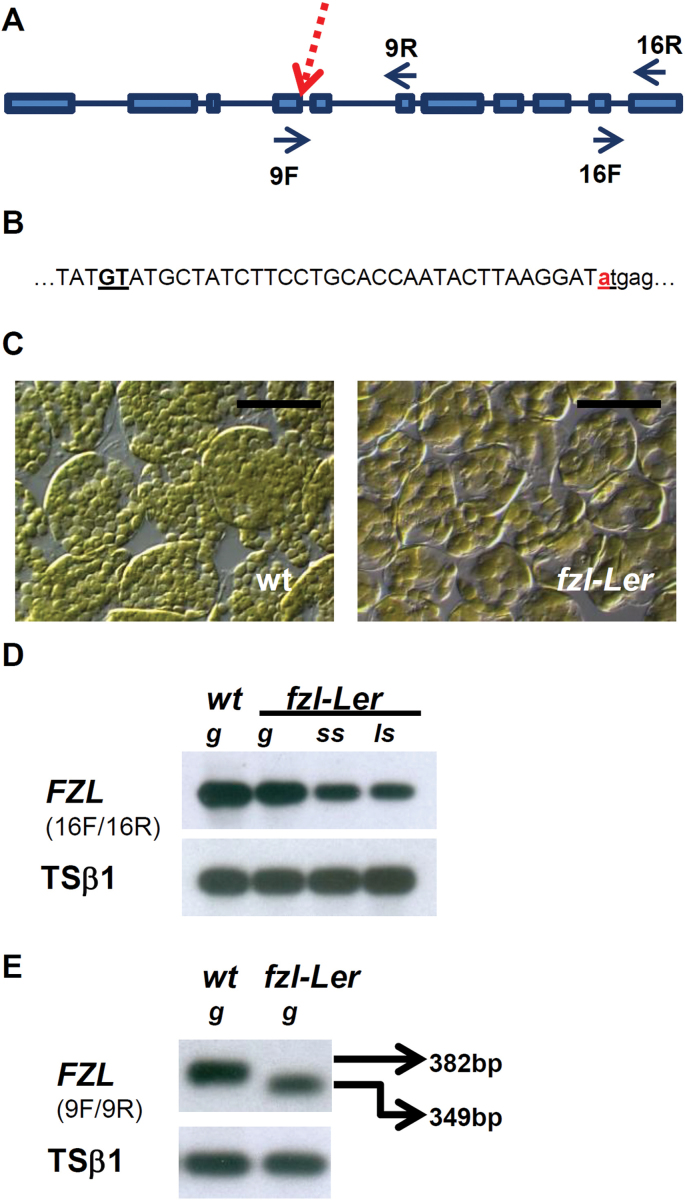
Molecular characterization of the *fzl-*L*er* mutation. (A) Schematic representation of the *FZL* gene. Boxes indicate exons, lines indicate introns, the dotted arrow indicates the site of *fzl-*L*er* mutation (first base of fourth intron), and arrows indicate the positions of the primers used for the RT–PCR analysis represented in D (primers 16F/16R) and E (primers 9F/9R). (B) Partial genomic sequence of the fourth exon (upper case letters) and fourth intron (lower case letters): the mutated base (a instead of the wild-type g) is indicated in red, and the activated cryptic site is underlined. (C) Chloroplast morphology of mesophyll cells of 3-week-old wild-type and *fzl-*L*er* plant leaves. Bars=50 µm. (D) RT–PCR analysis of *FZL* gene expression in wild-type and *fzl-*L*er* plants, using the primers FZL-16F/16R. (E) Analysis by RT–PCR of splicing of the fourth intron. Using the primers 9F/9R, a PCR product from the wild-type allele of 382bp is amplified and then sequenced, while from the *fzl-*L*er* allele a PCR product of 349bp is amplified and then sequenced. g, green leaves; ss, leaves with small chlorotic spots; ls, leaves with large chlorotic spots.

Massive parallel DNA sequencing was performed of mutant and L*er* plants generating 33 792 085 and 33 337 253 paired-end reads of 75–100bp, respectively. The alignment of the genomic sequence obtained from mutant and L*er* plant revealed a mutation (G→A) in the 5′ splice site of the fourth intron of the *FZL* gene (Ossowski *et al*., 2008), encoding a plant-specific member of the dynamin superfamily of membrane-remodelling GTPases, playing a unique role in the determination of chloroplast and thylakoid morphology (Gao *et al*., 2006) (Fig. 2A, B).

Because of the role played by *FZL*, the chloroplast structure was analysed in the isolated mutant and an alteration in the chloroplast morphology was found, very similar to that reported for the previously characterized T-DNA insertional mutants in the *FZL* gene (lines Salk_033745 and Salk_009051) (Gao *et al*., 2006) (Fig. 2C). The alteration of chloroplast morphology was already present very early during leaf development (12-day-old leaves) and the same level of alteration was present in 3-week-old leaves with or without chlorotic spots, suggesting the involvement of a developmental signal for HR cell death activation in *fzl* mutants.

Surprisingly the *fzl* mutants described by Gao and colleagues did not display a lesion mimic phenotype, but were characterized by delayed flowering and pale green leaves. Supposing that, together with the different environmental conditions, the different ecotypes in which these mutations were isolated (Columbia for the T-DNA insertional mutants, Landsberg *erecta* for the LMM) could account for the different phenotypes displayed, the LMM that was isolated is referred to as *fzl*-L*er* and the T-DNA insertional mutant is referred to as *fzl-*Col.

### Expression analysis of the FZL gene

The expression profile of the *FZL* gene was analysed in 3-week-old mutant and wild-type leaves. The RT–PCR analysis showed a decrease of *FZL* expression in the *fzl-*L*er* mutant (Fig. 2D), in agreement with the previously reported effect of mutations in the 5′ intron splicing site (Brown *et al*., 1996).

The *fzl-*L*er* cDNA was then analysed by sequencing the region amplified with the primers FZL9F/9R, to check if the mutation interferes with the correct splicing of the fourth intron. It was found that this mutation caused the activation of the next upstream cryptic splicing site in the fourth exon, thus causing the deletion of 33 nucleotides in the mature RNA (Fig. 2E) and the loss of 11 amino acids in the FZL protein. The deleted amino acids belong to the predicted domain with GTPase activity, where the deletion of the conserved Lys362 has been shown to modify the localization of *FZL*, with the consequent loss of function of the FZL protein (Gao *et al*., 2006); therefore, the molecular alteration found in the *fzl-*L*er* mutant is compatible with the severe phenotype observed.

### Genetic complementation of the fzl-Ler mutation.

**Fig. 3.**
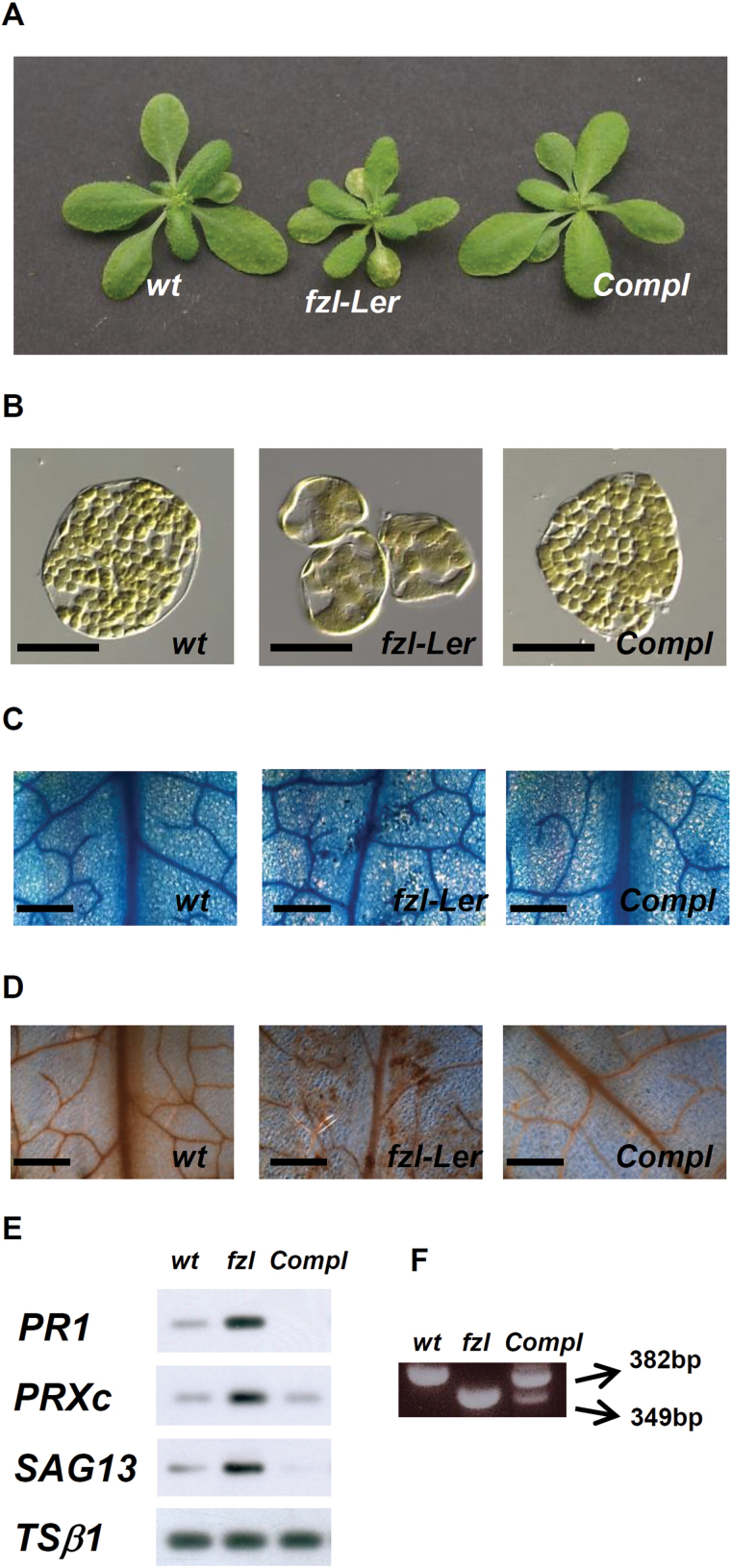
Genetic complementation of the *fzl-*L*er* phenotype. Phenotype (A), chloroplast morphology (B), Evan’s blue (C), and DAB (D) staining of 3-week-old plants of the wild type, *fzl-*L*er*, and *fzl-*L*er* complemented with the wild-type sequence of the *FZL* gene (Compl). Bars in B=50 µm; in C and D=500 µm. (E) Expression analysis of a group of defence-associated genes in 3-week-old plants of the wild type, *fzl-*L*er*, and *fzl-*L*er* complemented with the wild-type sequence of the *FZL* gene (Compl). (F) PCR analysis of the *FZL* transcript in wild-type, *fzl-*L*er*, and complemented plants (Compl). The primers used (3160-9F/10R) allowed the amplification of two distinct PCR products of 382bp and 349bp corresponding to the wild-type and the *fzl-*L*er* allele, respectively.

To confirm that the mutation in the *FZ*L gene was responsible for the mutant phenotype observed, mutant plants were transformed with the complete *FZL* genomic sequence. The phenotype of transformed plants was indistinguishable from the wild type at the macroscopic (no chlorotic lesions were present) (Fig. 3A), microscopic (no alterations in chloroplast morphology) (Fig. 3B), histochemical (no cell death and ROS accumulation) (Fig. 3C, D), and molecular level (no induction of disease response genes) (Fig. 3E), indicating that the mutant phenotype was associated with the mutation of the *FZL* gene.

Using PCR analysis, it was also checked whether the fourth intron was spliced correctly, and in the complemented plants, as expected, two different mature RNAs were found, the wild-type form, generated by correct splicing, and corresponding to a PCR product of 382bp, and the mutated form produced by the activation of the cryptic site of splicing and corresponding to a PCR product of 349bp (Fig. 3F).

### Effect of the environment on the fzl phenotype

**Fig. 4.**
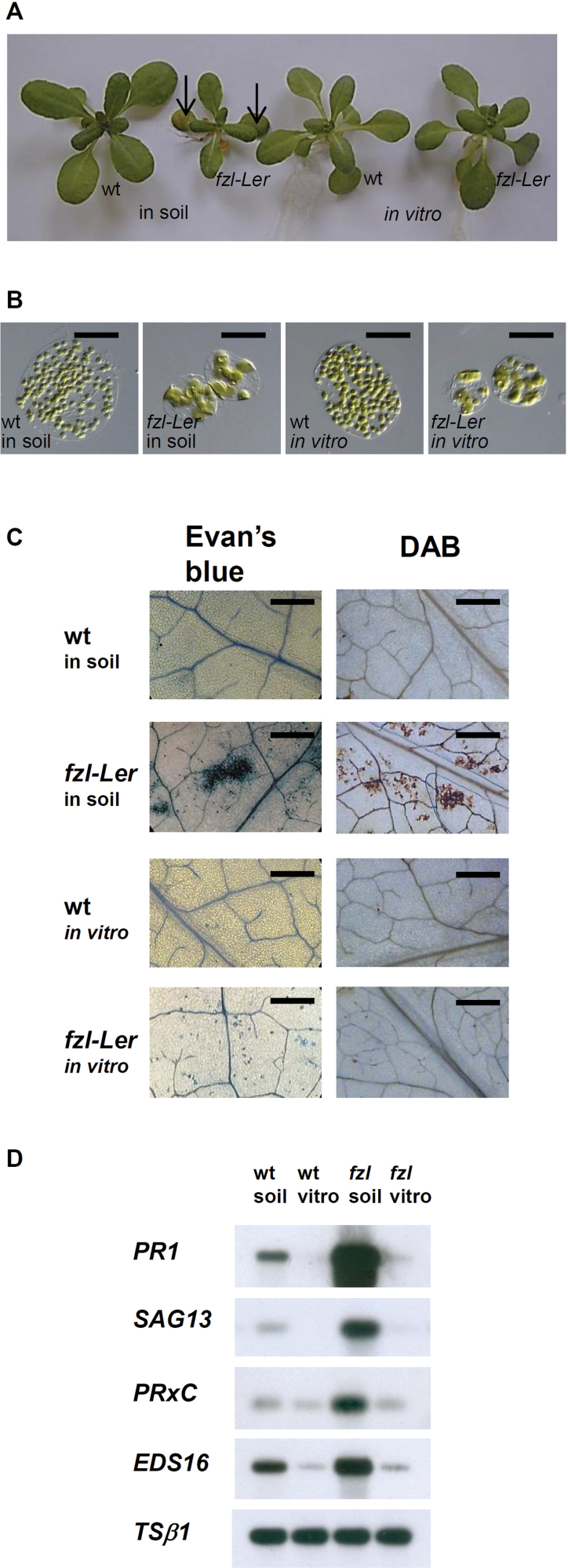
Effect of environmental conditions on the *fzl-*L*er* phenotype. Phenotype (A), chloroplast morphology of mesophyll cells (B), Evans’s blue and DAB staining (C), and expression analysis (D) of 3-week-old wild-type and *fzl-*L*er* plants grown in soil or *in vitro*. In A, arrows indicate the lesions present on *fzl-*L*er* rosette leaves. Bars in B=50 µm, in C=500 µm.

One of the characteristics shared by the LMMs described in the literature is the effect of environmental conditions in modulating the appearance of the lesions. The effect of growth under *in vitro* conditions (Brodersen *et al*., 2002; Lorrain *et al*., 2004) on the *fzl-*L*er* phenotype was analysed, and it was found that *fzl-*L*er* seedlings appeared indistinguishable from the wild type (Fig. 4A). Nevertheless microscopic analysis revealed that the chloroplasts of mutant plants grown *in vitro* showed the same morphological alterations observed in *fzl-*L*er* plants grown in soil (Fig. 4B). The expression of some of the previously analysed biochemical and molecular markers associated with HR was also checked, and while in *fzl-*L*er* mutants grown *in vitro* no DAB staining was detected, the Evan’s blue staining revealed the presence of some cell death (Fig. 4C). No significant difference was detected between wild-type and mutant leaves grown *in vitro* regarding the expression of the genes *PR1*, *PRXc*, *SAG13*, and *EDS16* (Fig. 4D).

**Fig. 5.**
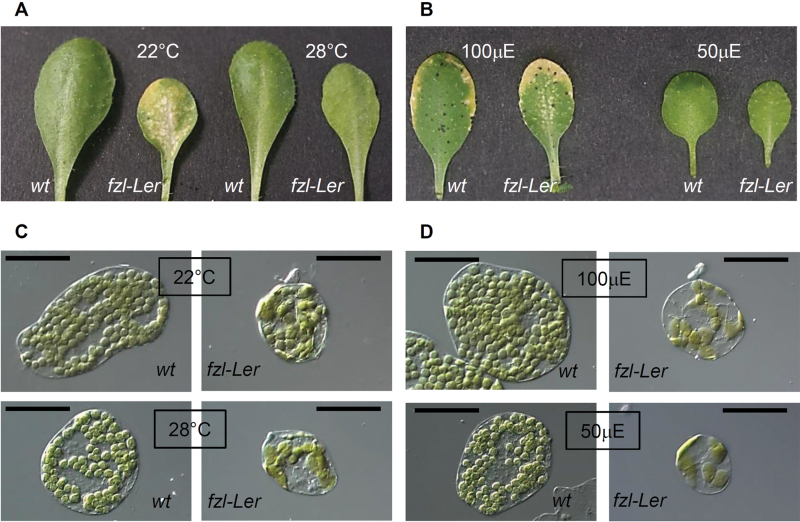
Effect of high temperature (28 °C) and low light intensity (50 µE) on the *fzl-*L*er* phenotype. Phenotype of 4-week-old leaves of the wild type and *fzl-*L*er* grown at 22 °C or 28 °C (A) and at a light intensity of 100 µE or 50 µE (B). Chloroplast morphology of mesophyll cells of 3-week-old wild-type or *fzl*-L*er* plants grown at 22 °C or 28 °C (C) and at a light intensity of 100 µE or 50 µE (D). Bars=50 µm.

Supposing that temperature, light, humidity and nutrient availability are the conditions that, in addition to the sterility, constitute the difference between *in vitro* and soil growth, these different conditions were tested one by one. The conditions that, at least partially, were able to suppress the mutant phenotype were growth at high temperature and at low light intensity (Fig. 5). In fact, when the plants were grown in soil at 28 °C or at a light intensity of 50 µE, the mutant leaves displayed a pale green colour instead of the typical chlorotic spots (Fig. 5A, B) even though histological analysis revealed that the chloroplasts still showed the morphological alterations observed in mutant plants grown under standard conditions (Fig. 5C, D).

### Effect of the genetic background on the fzl phenotype

**Fig. 6.**
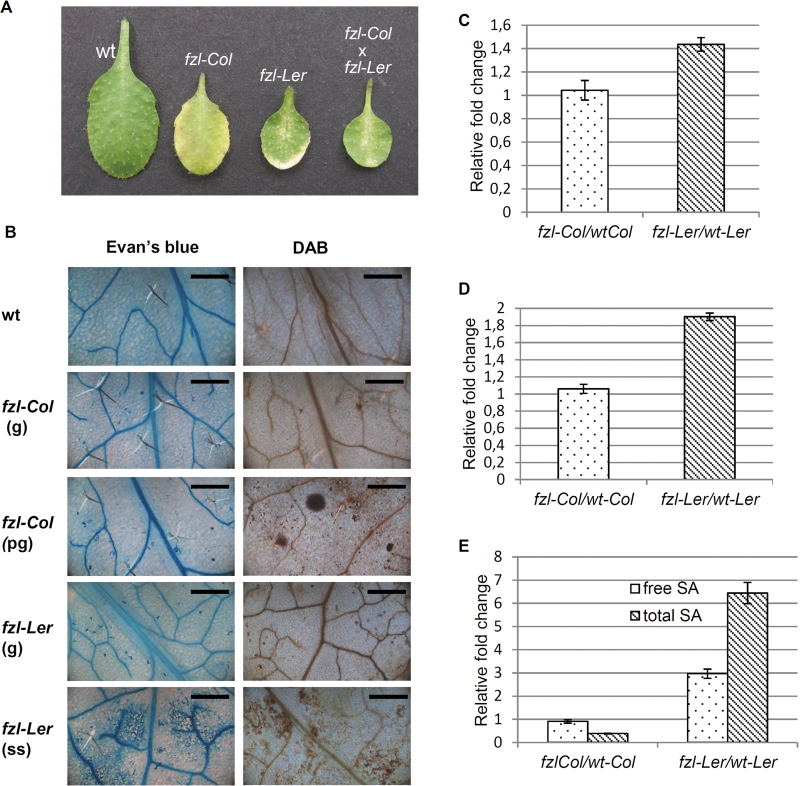
Comparison of the *fzl* mutant phenotype in the two ecotypes Columbia (*fzl*-Col) and Landsberg (*fzl*-L*er*). (A) Phenotype of 3-week-old leaves, from left to right: wild type, *fzl*-Col, *fzl*-L*er*, and F_1_ progeny obtained by crossing *fzl*-Col×*fzl*-L*er*. (B) Evan’s blue and DAB staining on 3-week-old leaves of the wild type, *fzl*-Col, and *fzl*-L*er*. g, green leaves; pg, pale green leaves; ss, leaves with small spots. Bars=500 µm. (C) Cell death quantification by electrolyte leakage measurement. Data reported refer to the time point 30min, but a similar trend was observed at the successive time points analysed (data not shown). Values are expressed as fold change relative to the wild type and are the mean of three replicates. Bars represent the standard error. (D) H_2_O_2_ content quantified as fold change relative to the wild type. Values are the mean of three replicates. Bars represent the standard error. (E) free and total SA measurement. Values are the mean of three replicates. Bars represent the standard error.

To understand the different effects of the *fzl* mutation on the Columbia/Landsberg ecotypes, the phenotype (Fig. 6A), the accumulation of the biochemical markers associated with HR (Fig. 6B–D), and the levels of free and total SA in *fzl*-Col mutants versus *fzl*-L*er* mutants (Fig 6E) were compared.

The histochemical results showed that while in wild type leaf, H_2_O_2_ and dead cells are never present, in *fzl*-Col mutants these markers can be detected, and their level increased with the severity of the phenotype, even if the higher levels displayed in *fzl*-L*er* mutants are never reached in *fzl*-Col mutants (Fig. 6B).

The quantification assays of H_2_O_2_ and cell death in *fzl* mutants confirmed the high level of both in the *fzl-*L*er* mutant (80% and 40% higher, respectively, than in the relative wild type) while only a very small or no increase in these parameters was observed for the *fzl*-Col mutant in comparison with the control. A similar result was obtained with free and total SA quantification: while the *fzl-*L*er* mutant accumulated a higher level (3- and 6-fold, respectively) of these molecules in comparison with the wild type, no difference (free SA) or a decrease (total SA) were detected when comparing the *fzl*-Col mutant with the wild type.

The *fzl-*L*er* mutant was also crossed with *fzl*-Col mutants, and it was found that in the F_1_, the mutant phenotype was characterized by the presence of chlorotic spots very similar to those shown by the *fzl-*L*er* mutant (Fig. 6A).

### Analysis of double mutants

To check which among the signalling pathways known to be involved in HR regulation and execution are required for the determination of the *fzl-*L*er* mutant phenotype, the *fzl*-L*er* mutant was crossed with mutants altered in these pathways.

**Fig. 7.**
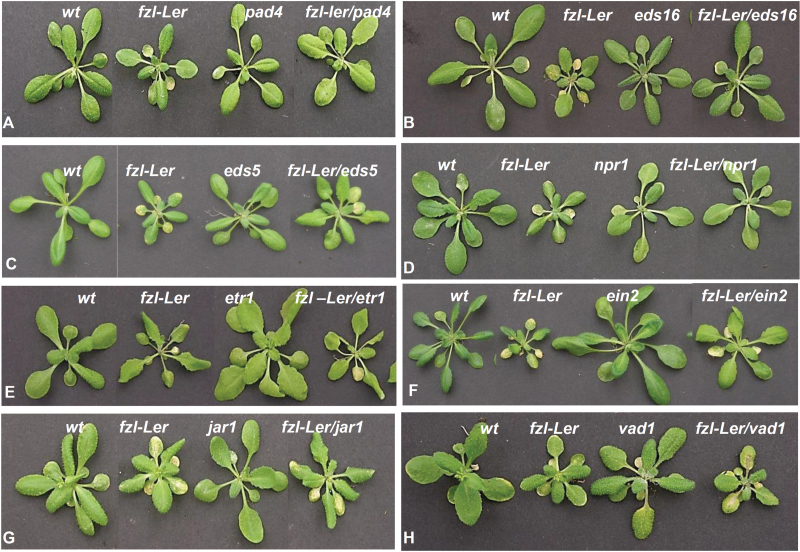
Phenotypic analysis of 3- to 4-week-old double mutants obtained by crossing *fzl-*L*er* with mutants in the signalling pathways activated in HR. The L*er* ecotype is used as the wild type.

**Fig. 8.**
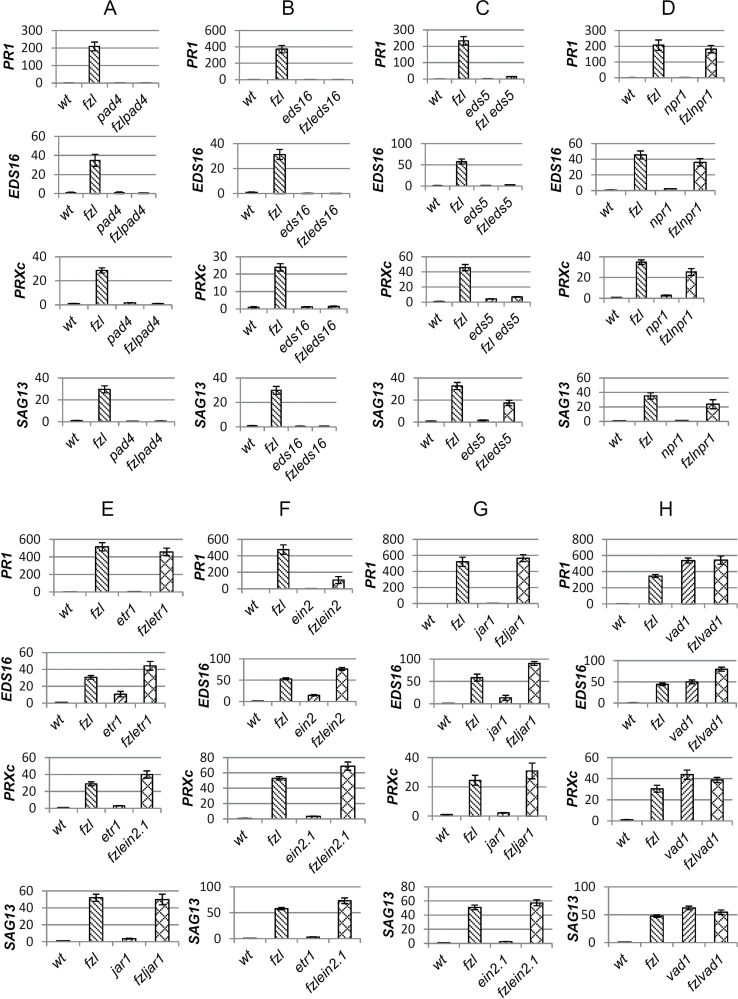
Expression analysis by real-time RT-PCR of a group of defence-associated genes in 3- to 4-week-old wild-type, *fzl-*L*er*, single mutant, and double mutant plants whose phenotype is represented in Fig. 7. The L*er* ecotype is used as the wild type. On the *y*-axis is represented the relative expression level of the genes analysed using Tsβ1 as the endogenous control. Bars represent the standard errors of measurements performed in triplicate.

The *fzl-*L*er* mutant was in the L*er* background while all the other signalling mutants used in this analysis were in the Col background, but it had already been verified that the *fzl-*L*er* mutation was still perfectly recognizable in the mixed background L*er*/Col when the segregant population for the positional cloning of the *fzl-*L*er* mutation was generated. Double mutants, single mutants, and the wild type were then compared by the analysis of the phenotype (Fig. 7) and the expression level of some defence-related genes previously used to characterize the single mutant *fzl-*L*er* (Fig. 8).

The double mutant *fzl-*L*er*/*pad4* showed no lesion formation; its size was comparable with that of the single *pad4* mutant (Fig. 7A), and no induction of the defence-associated genes analysed was detected (Fig. 8A), suggesting a role for PAD4 (Wiermer *et al*., 2005) in both the defence response and cell death activation.

To determine the role of SA in lesion formation and defence response activation, *fzl-*L*er* plants were crossed with the two mutants *eds16* and *eds5* (Wildermuth *et al*., 2001; Nawrath *et al*., 2002). In *fzl-*L*er*/*eds16* plants, the *fzl-*L*er* phenotype was completely suppressed (Fig. 7B) and the expression of defence-related genes was not induced (Fig. 8B), while in *fzl-Ler*/*eds5* plants the lesion formation was only delayed, the lesion size reduced (Fig. 7C), and the defence-related genes were induced to a level similar to that observed in the single *fzl-*L*er* mutant (Fig. 8C).

Under standard growth conditions, the *fzl-*L*er*/*npr1* mutant showed a wild-type phenotype (Fig. 7D) (even though sometimes in suboptimal environmental conditions, such as high temperature and low water availability, some lesions appeared), and expression analysis showed enhanced expression of defence-related genes (Fig. 8D).

To check the involvement of the ethylene–JA pathways in *fzl*-L*er* lesion formation, the double mutants *fzl-*L*er*/*etr1*, *fzl-*L*er*/*ein2*, and *fzl-*L*er*/*jar1* were generated. In all these double mutants, the timing of the appearance and the development of the lesions was similar to that observed in the single mutant *fzl*-L*er* (Fig. 7E–G), and the expression level of defence-associated genes was the same as that shown by the *fzl-*L*er* single mutant (Fig. 8E–G).

The *fzl-*L*er* mutant was also crossed with the propagative LMM *vad1* (vascular associated death1) (Lorrain *et al*., 2004; Bouchez *et al*., 2007) to analyse the effect of the interaction of two different LMMs. In *fzl-*L*er*/*vad1* plants, the mutant phenotype was more severe than that in the two single mutants, the lesions appeared earlier and their propagation was more rapid (Fig. 7H), while the expression level of the defence-associated genes was similar in the two single and in the double mutant (Fig. 8H).

## Discussion

The characterization of an *Arabidopsis* mutant which displayed chlorotic lesions on rosette leaves and reduced size caused by a single recessive mutation in the *FZL* gene, encoding a GTPase involved in the determination of thylakoid and chloroplast morphology (Gao *et al*., 2006), is reported. This mutant was isolated in the L*er* ecotype and, because of the different phenotype shown by the previously described *fzl* insertional mutants, isolated in the Col background (Gao *et al*., 2006), the mutant was named *fzl*-L*er*.

Histochemical analysis showed the presence, in and around the lesions, of the biochemical markers typically associated with the activation of the defence programme, and expression analysis revealed the constitutive activation of genes known to be markers of HR. These traits are generally associated with the LMMs previously described in the literature (reviewed by Lorrain *et al*., 2003), so it is suggested that *fzl-*L*er* is a member of this class of mutants.

The analysis of double mutants showed that the loss of *EDS16*, *EDS5*, and *PAD4* functions resulted in the reduction/absence of both the lesions and the defence programme activation, suggesting a central role for SA in the HR cell death process.

The absence of lesions and the high level of expression of the HR marker genes in the double mutant *fzl-*L*er*/*npr1* suggested that in the *fzl-*L*er* mutant cell death activation is NPR1 dependent, while an NPR1-independent pathway is involved in defence gene activation. A dual role for *NPR1* has also been reported previously for the mutant *vad1* (Lorrain *et al*., 2004); in this case, NPR1 function was required for defence activation but not for cell death.

The *fzl-*L*er* mutant was crossed not only with mutants altered in defence signalling pathways but also with the propagative LMM *vad1* (Lorrain *et al*., 2004; Bouchez *et al*., 2007), with the aim of checking the existence of cross-talk between the signalling pathways activated in these two mutants that look very similar in some aspects. Both are propagative LMMs, and both require SA and ROS in the signalling cascade leading to the mutant phenotype. However, for other traits, they look the opposite of one another: *vad1* lesions are associated with the vascular system, whereas the *fzl-*L*er* lesions are close to the veins that however are never affected by cell death; and *vad1* phenotypes require the activation of the ethylene pathway, while *fzl-*L*er* phenotypes are completely independent from this signalling.

The two mutations *fzl-*L*er* and *vad1* resulted in the additive phenotype of the double mutant *fzl-*L*er*/*vad1*, thus suggesting that the two single mutants are altered in different signalling pathways, but are acting additively in the double mutant to activate defence responses and HR cell death.

It has been previously hypothesized that some LMMs may derive from a metabolic imbalance, as in the case of organelle malfunctioning (Mur *et al*., 2008). In the case of the *fzl-*L*er* mutant, it was shown that a mutation causing the alteration of chloroplast morphology is linked to an LMM phenotype. More specifically, in the *fzl*-L*er* mutant, a branch of the HR signalling pathway is constitutively activated: this is SA/ROS dependent, requiring the genes *EDS5*, *EDS16*, *PAD4*, and *NPR1*, cross-talking with the senescence signalling, and is independent from the ethylene–JA pathway.

Since the first reports on LMMs (Walbot *et al*., 1983), but also recently (Yamaguchi *et al*., 2012; Wituszynska *et al*., 2013), it has been highlighted that not only environmental conditions (temperature, light, humidity, etc.) but also the genetic background is an important factor influencing the LMM phenotype. It is therefore not surprising that the *fzl* mutation in the Columbia ecotype was reported to cause pale leaves and delayed flowering (Gao *et al*., 2006) while it was found that in the L*er* background the loss of function of the *FZL* gene determined a typical LMM phenotype.

The observation that the phenotype displayed by the *fzl-*L*er* mutant partially recovered by high temperature or low light treatments was very similar to the *fzl*-Col phenotype, suggested that the *fzl-*Col mutant could be seen as the ‘mild’ version of the typical LMM phenotype shown by the *fzl-*L*er*. This difference can be due to both the different type of mutation present in the *FZL* gene in the two backgrounds and to the natural variation existing among these ecotypes that has been previously reported to account for differences in the lipid composition of thylakoid membranes (Yin *et al*., 2012), ROS-scavenging activities (Nagata *et al*., 2003), *R* genes (Tahir *et al*., 2013), and susceptibility to, and symptoms after, bacterial (Buell and Sommerville, 1997; Godiard *et al*., 2003), fungal (Denby *et al*., 2004; Chen *et al*., 2006; Birker *et al*., 2009), and viral infections (Kaneko *et al*., 2004; Sicard *et al*., 2008).

For a more complete dissection of the effect of the genetic background on the LMM phenotype it will also be interesting to analyse the effect of temperature and light intensity on *fzl*-Col mutants.

DAB staining showed H_2_O_2_ accumulation in *fzl-*L*er* green leaves, at the sites where subsequently the lesions will appear, thus suggesting a central role for ROS signalling in cell death initiation in *fzl-*L*er* mutants. In wild type non-stressed cells, ROS are produced as normal by-products of aerobic metabolism; to prevent ROS accumulation and the consequent oxidative cell damage, the equilibrium between ROS production and scavenging is strictly regulated (Apel and Hirt, 2004).

Moreover it is known that during senescence the tightly regulated chloroplast dismantling process has the function of avoiding the release of the potentially phototoxic chlorophyll, thus suggesting that in plants, cell death can be regulated through the control of chloroplast integrity (Gray *et al*., 2002).

Stress conditions, both biotic and abiotic, cause increased ROS production (Møller and Sweetlove, 2010) and after pathogen attack their role in the defence response is due not only to their effect as anti-microbial compounds but also to their role as signalling molecules leading to HR (Jabs *et al*., 1996).

A mechanism for chloroplast generation of ROS during HR is presented in the model proposed by Zurbriggen and collaborators, in which chloroplasts are the initial source of ROS after pathogen attack through the shutdown of electron utilization in the chloroplast stroma, determining the over-reduction of the photosynthetic electron transport chain and EEE in the thylakoids: the signalling then is spread, by the activation of NADPH oxidases, to the apoplast and to the adjacent cells, leading to HR cell death (Zurbriggen *et al*., 2009, 2010).

Alterations in both biosynthesis and breakdown of chlorophyll pathways have been reported to generate the accumulation of phototoxic intermediates, resulting in light-dependent lesion mimic phenotypes (Kruse *et al*., 1995; Hu *et al*., 1998; Ishikawa *et al*., 2001; Mach *et al*., 2001; Pružinská *et al*., 2003). Interestingly, while mutations in the biosynthetic pathway generally result in the initiative lesion mimic phenotype (Hu *et al*., 1998; Ishikawa *et al*., 2001), mutations in the catabolic pathway are associated with propagative phenotypes (Mach *et al*., 2001; Pružinská *et al*., 2003).

The *Arabidopsis* propagative LMM *acd1* and its maize orthologue *lls1* are deficient in PAO (pheophorbide *a* oxygenase), the key enzyme of chlorophyll catabolism: these mutants accumulate the phototoxic chlorophyll catabolite pheide *a* that in a light-dependent manner allows the production of ROS, the presumed diffusible signal responsible for the spread of the lesions (Pružinská, *et al*., 2003). The first morphological alteration reported in *lls1* mutants is the loss of structural integrity of chloroplast and thylakoid membranes in mesophyll cells (Gray *et al*., 2002), which, by causing the leakage of phototoxic chlorophyll intermediates, determine the propagative cell death (Pružinská *et al*., 2003).

However, alterations in chlorophyll metabolism cannot account for all the events of the release of phototoxic compounds from the chloroplast. Chlorophyll degradation occurs during all the phases of the life cycle of the plant for the normal turnover of chlorophyll, not only during senescence; furthermore, both biotic and abiotic stress can damage plant cells, resulting in chlorophyll release from the thylakoid membranes (Takamiya *et al*., 2000). Moreover, it is known that damaged chloroplasts not only activate a retrograde signalling to down-regulate the nuclear genes encoding the photosynthetic apparatus, but also, through the accumulation of ^1^O_2_^–^, are able to trigger cell death signalling pathways (Galvez-Valdivieso and Mullineaux, 2010).

Two recent studies (Kim *et al*., 2012; Nomura *et al*., 2012) suggested a role for the chloroplast in the signalling cascade leading to PCD, while Noshi and colleagues demonstrated that chloroplastic H_2_O_2_ enhances the levels of SA and the response to SA (Noshi *et al*., 2012).

Recently it has also been reported that another mutation in a gene encoding a chloroplast membrane protein (*AtLrgB*) resulted in an LMM phenotype (Yamaguchi *et al*., 2012; Yang *et al*., 2012). The mutant phenotypes are completely rescued when the plants are grown under continuous light; thus, the authors suggested that the *AtLrgB* gene, in contrast to *fzl-L*er, is not essential for chloroplast development (Yamaguchi *et al*., 2012).

The hypothesis from the present findings is that the loss of integrity of the chloroplast membrane system observed in the *fzl-*L*er* mutant, determining the interruption of the electron transport chain and the release of chlorophyll phototoxic intermediates/catabolites, might be responsible for the release of a ROS-based signalling, which, overlapping with the signalling generated by a pathogen attack, turned on the HR signalling cascade, resulting in the activation of defence programmes and cell death. This is in agreement with the observations that the *fzl-*L*er* phenotype is partially light dependent, as reported for other propagative LMMs in which the HR signalling is triggered by the photoactivation of chlorophyll catabolites (Mach *et al*., 2001; Pružinská *et al*., 2003), and may be partially reversed by high temperatures, known to down-regulate ROS production by the activation of scavenging enzymes during HR (Kiraly *et al*., 2008).

Further work will be focused on the identification of the chloroplast-derived signal generated in *fzl-*L*er* mutants and on its specific role in HR cascade activation.

The *fzl-*L*er* mutation also appears to be a useful tool with potential to unravel the mechanisms underlying two agronomic traits of fundamental importance in breeding programmes aimed at enhancing plant productivity, namely pathogen resistance and the control of the timing of leaf senescence.

## Supplementary data

Supplementary data are available at *JXB* online.

Table S1. Oligonucleotides used for RT–PCR analysis.

Table S2. Oligonucleotides used for real-tme RT–PCR analysis.

Supplementary Data
