# Supplementary material for: A mutation in the FZL gene of Arabidopsis causing alteration in chloroplast morphology results in a lesion mimic phenotype
Source: J Exp Bot. 2013 Aug 19;64(14):4313–28. doi: 10.1093/jxb/ert237 (PMC3808314; doi:10.1093/jxb/ert237)
Supplement: Supplementary Data [file supp_64_14_4313__index.html]

A mutation in the FZL gene of Arabidopsis causing alteration in chloroplast morphology results in a lesion mimic phenotype — A mutation in the FZL gene of Arabidopsis causing alteration in chloroplast morphology results in a lesion mimic phenotype — Supplementary Data 

# A mutation in the *FZL* gene of *Arabidopsis* causing alteration in chloroplast morphology results in a lesion mimic phenotype

## 

Data files

**Files in this Data Supplement:**

- Supplementary Data - Supplementary Data
