# Supplementary material for: A mutation in the FZL gene of Arabidopsis causing alteration in chloroplast morphology results in a lesion mimic phenotype
Source: J Exp Bot. 2013 Aug 19;64(14):4313–28. doi: 10.1093/jxb/ert237 (PMC3808314; doi:10.1093/jxb/ert237)
Supplement: Supplementary Data [file supp_ert237_jexbot099465_file001.pdf]

"A mutation in the *FZL* gene of Arabidopsis causing alteration in chloroplast morphology results in a lesion mimic phenotype".

Michela Landoni, Alessandra De Francesco, Silvia Bellatti, Massimo Delledonne, Alberto Ferrarini, Luca Venturini, Roberto Pilu and Chiara Tonelli

Supplemental Table S1. Oligonucleotide primers used for RT-PCR analysis.

Gene name	Primer	Sequence
Tsβ1	Tsβ1F	5'-CTCATGGCCGCCGGATCTTGA-3'
	Tsβ1R	5'-CTTGTCTCTCCATATCTTGAGCA -3'
FZL	3160 16F	5'- GTGGGCTTGGAGCTGCAG -3'
	3160 16R	5'- GTCGGAACTGGCAGGTATTATTC -3'
	3160 9F	5'-GCACTTCTTGGGAAGAGATACCTG-3'
	3160 9R	5'- CTCACGAGCATCACGATAGATATC -3'
PR1	FPR1	5'-ATGAATTTTACTGGCTATTCTCGATTTTTA -3'
	RPR1	5'-TTAGTATGGCTTCTCGTTCACATAATT-3'
Pal1	FPal1	5'-CTTACTACTGGAGTCAATGGTGAG-3'
	RPal1	5'-TCCACTCGTTGAGATATTCCATC-3'
PRXc	FPRXc	5'-CAGAAGGACGATCGAGACCAA-3'
	RPRXc	5'-TGACGCATCCATCTTGTTAGACA-3'
PDF1.2	FPDF1.2	5'-TTAACATGGGACGTAACAGATACACTT-3'
	RPDF1.2	5'-TTAACATGGGACGTAACAGATACACTT-3'
SAG12	FSAG12	5'-GGCACATCGAGTGGATGACT-3'

	RSAG12	5’-TGCCTTCATCAGTGCTTGCT-3’
SAG13	FSAG13	5’-GGCACATCGAGTGGATGACT-3’
	RSAG13	5’-CCACAAGCGGTGAGACTTCA-3’
PAD3	PAD3F	5’-ATGTCGGTTTTCCTCTGTTTCCT-3’
	PAD3R	5’-CCTTCTTTCAAGTTCTTCACCACTG-3’
Athsr3	Athsr3F2	5’-GTTGGCCTACCGATTTGTTCTTCCAG-3’
	Athsr3R2	5’-ACGGTGATGAGCCTCGTCAGCA-3’
EDS1	EDS1F2	5’-CAAGGAAGAAGCAGGAGCAGTCGTA-3’
	EDS1R2	5’-GTACCGGCGGGTCTATAAGGACT-3’
EDS16	EDS16F	5’- CCGACATTGATCCCATTGCTTGGCTA-3’
	EDS16R	5’- CTTCCTTCGTGAGTTTCCCTGCCAATT-3’

Supplemental table S2. Oligonucleotide primers used for RealTime RT-PCR analysis.

Gene name	Primer	Sequence
Actin	AtACT2F	5'-TGCTTCTCCATTTGTTTGTTTC-3'
	AtACT2R	5'-GGCATCAATTCGATCACTCA-3'
PR1	PR1F2	5’-GGAGCTACGCAGAACAACTAAGA-3’
	PR1R2	5’-CCACCATTGTTACACCTCACTTT-3’
PRXc	PRXcF-2	5’-CCTCCTGCCAAAGTGACAGAT-3’
	PRXcR-2	5’-TGACGCATCCATCTTGTTAGACA-3’
SAG13	SAG13-1F-2	5’-TTCGTCCTCCTCGGTATTGGG-3’
	SAG13-1R-2	5’-GTGAGAGATGGAAAGCTGACTCGA-3’
EDS16	EDS16-2F	5’- GATAGAGGAATGTATGCGGGAC-3’
	EDS16-2R	5’- GGTAACAGAGAACCATGATGTCAC-3’
